# *ZDHHC8 *as a candidate gene for schizophrenia: Analysis of a putative functional intronic marker in case-control and family-based association studies

**DOI:** 10.1186/1471-244X-5-35

**Published:** 2005-10-14

**Authors:** Thomas Faul, Micha Gawlik, Martin Bauer, Sven Jung, Bruno Pfuhlmann, Burkhard Jabs, Michael Knapp, Gerald Stöber

**Affiliations:** 1 Department of Psychiatry and Psychotherapy, University of Würzburg, Füchsleinstraße 15, 97080 Würzburg, Germany; 2 Department of Forensic Medicine, University of Würzburg, Lindleinstraße 15, 97080 Würzburg, Germany; 3 Institute of Medical Biometry, Informatics and Epidemiology, University of Bonn, Sigmund-Freud-Str. 25, 53105 Bonn, Germany

## Abstract

**Background:**

The chromosome 22q11 region is proposed as a major candidate locus for susceptibility genes to schizophrenia. Recently, the gene ZDHHC8 encoding a putative palmitoyltransferase at 22q11 was proposed to increase liability to schizophrenia based on both animal models and human association studies by significant over-transmission of allele rs175174A in female, but not male subjects with schizophrenia.

**Methods:**

Given the genetic complexity of schizophrenia and the potential genetic heterogeneity in different populations, we examined rs175174 in 204 German proband-parent triads and in an independent case-control study (schizophrenic cases: n = 433; controls: n = 186).

**Results:**

In the triads heterozygous parents transmitted allele G preferentially to females, and allele A to males (heterogeneity χ^2 ^= 4.43; *p *= 0.035). The case-control sample provided no further evidence for overall or gender-specific effects regarding allele and genotype frequency distributions.

**Conclusion:**

The findings on rs175174 at ZDHHC8 are still far from being conclusive, but evidence for sexual dimorphism is moderate, and our data do not support a significant genetic contribution of rs175174 to the aetiopathogenesis of schizophrenia.

## Background

Systematic approaches designed to identify schizophrenia susceptibility genes have implicated the chromosome 22q11 microdeletion region as a promising hot-spot [1,2]. Recently, Mukai and colleagues [3] proposed a gender-specific effect of the ZDHHC8 gene at 22q11 to liability to schizophrenia based on animal models followed by human association studies. ZDHHC8 is a brain expressed putative palmitoyltransferase of 765 amino acids and may, thus, be involved in synaptic transmission and posttranslational modification of yet unidentified substrates. The ZDHHC8 gene consists of 11 exons spanning ~16 kb on genomic DNA (; OMIM: *608784). A single nucleotide polymorphism (SNP), rs175174 at intron 4, provided the highest significance from the entire 22q11 locus [4], and cell experiments gave evidence that rs175174 (A/G) may modify ZDHHC8 expression by causing imperfect splicing, intron retention and reduced enzyme activity. Zdhhc8 knockout mice had sexually dimorphic deficits in prepulse inhibition and other features proposed to mimic schizophrenia in mice. Subsequently, fitting into the animal model analysis of 389 proband-parent trios showed significant over-transmission of allele A in female, but not male subjects with schizophrenia [3]. Studies aiming replication of the genetic evidence challenged the initial findings in populations of Asian or European origin either by a lack of association or by reporting the allele G over-transmitted to schizophrenia in a non-gender specific manner [5-7]. Given the genetic complexity of schizophrenia and the potential genetic heterogeneity in different populations as highlighted by the recent findings, we decided to conduct an association study on rs175174 in ZDHHC8 using both population- and family-based samples of an ethnically homogenous German population.

## Methods

The proband-parent trio sample encompassed 204 cases, who fulfilled diagnostic criteria of DSM IV for schizophrenia [8], and their biological parents. The index cases (136 males; 67%) had a mean age at onset of 23.8 years (SD 7.5) and an age at assessment of 32.4 years (SD 8.8). In 60 triads (29%) we found a parent (33 females) previously treated for psychosis. There was no evidence for bilineal transmission in the sample. The sample for the independent case-control association study involved 433 probands with schizophrenia (321 males; 74%). The probands had a mean age of first hospitalization at 28.9 years (SD 10.6), and an age at assessment of 39.2 (SD 13.5) years. In 15% (n = 64) a further first-degree relative had been recorded as hospitalized for schizophrenia (familial cases). Subjects fulfilled diagnosis of cycloid psychoses (n = 149), unsystematic (n = 155) and systematic schizophrenia (n = 129) according to differentiated psychopathology [9]. The probands were recruited at the Department of Psychiatry and Psychotherapy at the University of Würzburg. The 186 volunteer control subjects (105 males; 56%) were recruited from the blood donor centre at the University of Würzburg at a mean age of 30.0 years (SD 10.3). All subjects were unrelated and of German Caucasian descent. The Ethics Committee of the University of Würzburg had approved the study, and informed consent was obtained from all subjects.

PCR for allelic discrimination at SNP rs175174 (A/G) was performed in a final reaction volume of 10 μl containing 20 ng genomic DNA and 5 μl of 20× TaqMan^®^Universal PCR Master Mix (Applied Biosystems) and 0.5 μl of 20× TaqMan™ validated SNP genotyping assay including fluorescent tags specific for the wild type allele and the variant allele. Marker amplification was performed in microtiter plates on Biometra machines (Whatman). PCR amplification conditions were according to the facturer's recommendation [10 min at 95°C followed by 15 sec at 92°C and 60 sec at 60°C for 40 cycles]. Allelic discrimination with endpoint detection of fluorescence was performed at 60°C on an ABI prism 7000 sequence detection system followed by analysis with an appropriate software package (Applied Biosystems).

Fisher's exact and Armitage's trend test and were used to compare allelic and genotypic distributions between cases and controls. One-way ANOVA as implemented in the SAS was applied to compare the age-at-onset between genotypes. The data of the proband-parent triads were analyzed by the transmission-disequilibrium-test (TDT) [10]. Power calculations are based on the approximation described by Jackson et al. [11]; the exact test proposed by Weir [12] was applied for Hardy-Weinberg equilibrium.

## Results

In the sample of 204 triads, no transmission distortion was apparent in the whole sample (TDT = 0.13, *p *= 0.72; Table 1) and in the sub-samples of families with maternal affection (TDT = 0.13, *p *= 0.72) or paternal affection (TDT = 0.17, *p *= 0.68). Even after stratifying for gender of the proband, there was no significant over-transmission of an allele to males (TDT = 2.31, *p *= 0.13) and to females (TDT = 2.25, *p *= 0.14; Table 1). However, allele A is preferentially transmitted to males, whereas allele G is more often transmitted to females and this gender-related heterogeneity of allele transmission was significant (heterogeneity χ^2 ^= 4.43; *p *= 0.035).

**Table 1:** Transmission of marker rs 175174 A/G in 204 traids with schizophrenic psychoses

	total sample (N = 204)			females (N = 68)			males (N = 136)		
MT	AA	AG	GG	AA	AG	GG	AA	AG	GG

AAxAA	32	-	-	10	-	-	22	-	-
AAxAG	37	31	-	9	13	-	28	18	-
AAxGG	-	32	-	-	13	-	-	19	-
AGxAG	14	23	14	4	7	8	10	16	6
AGxGG	-	9	10	-	2	2	-	7	8
GGxGG	-	-	2	-	-	0	-	-	2
n	83	95	26	23	35	10	60	60	16
%	40.7	46.6	12.7	33.8	51.5	14.7	44.1	44.1	11.8
HWE p-value	0.37			0.78			0.37		
TDT	97 T/92 NT			26 T/38 NT			71 T/54 NT		
p-value	0.72			0.13			0.13		

Transmission from heterozygous parents: MT = Mating Type; HWE = Hardy-Weinberg-Equilibrium; TDT = transmission-disquilibrium test; T = transmitted, NT = Non-transmitted.

In our case-control panel of 619 individuals, allele and genotype frequencies were not significantly different between cases and controls, and there was no evidence for gender-specific effects (Table 2). The familial/sporadic distinction produced negative results as did a sub-classification according to differentiated psychopathology (data not shown). Finally, genotypes were not associated with different mean age-at-onset in both panels (ANOVA p = 0.066). Genotypes were in appropriate Hardy-Weinberg equilibrium (HWE), and the allele frequency of rs175174G was equal in the volunteer control sample (0.63) and the non-transmitted alleles in triads (0.63).

**Table 2:** Allele and genotype frequency at marker rs 175174 in schizophrenic psychoses

	Allele (%)			Genotypes (%)			
Samples	A	G	p-value	AA	AG	GG	p-value

total							
SCZ (N = 433)	527 (60.9)	339 (39.1)	0.48	154 (35.6)	219 (50.6)	60 (13.9)	0.43
CON (N = 186)	235 (63.2)	137 (36.8)		71 (38.2)	93 (50.0)	22 (11.8)	
females							
SCZ (N = 112)	125 (55.8)	99 (44.2)	0.25	30 (26.8)	65 (58.0)	17 (15.2)	0.20
CON (N = 81)	100 (61.7)	62 (38.3)		27 (33.3)	46 (56.8)	8 (9.9)	
males							
SCZ (N = 321)	402 (62.6)	240 (37.4)	0.68	124 (38.6)	154 (48.0)	43 (13.4)	0.66
CON (N = 105)	135 (64.3)	75 (35.7)		44 (41.9)	47 (44.8)	14 (13.3)	
family history							
sporadic SCZ (N = 369)	441 (59.8)	297 (40.2)	0.12	124 (33.6)	193 (52.3)	52 (14.1)	0.10
familial SCZ (N = 64)	86 (67.2)	42 (32.8)		30 (46.9)	26 (40.6)	8 (12.5)	

SCZ: probands with schizophrenia; CON: controls; p-values for alleles (Fisher's exact test) and genotypes (Armitage's trend test).

## Discussion

In independent case-control and proband-parent samples we attempted replication of a gender-specific transmission of allele rs175174 A to females at the ZDHHC8 gene locus. In contrast to the original report of Mukai and colleagues [3] we found moderate evidence at *p*-level 0.035 of a sex-related heterogeneity of allele transmission with preferential transmission of allele A to males and allele G to females. This observation remained the only signal for a genetic involvement of ZDHHC8 in schizophrenia from our samples. The preferential transmission of allele G corresponded partially to the report of Chen and colleagues [5] that allele G instead of A is preferentially transmitted to schizophrenic subjects, in opposite to the animal model and the initial genetic data from U.S. and Afrikaner pedigrees [3]. However, in the present study gender-specific effects were restricted to proband-parent triads, whereas one further study found non-gender related associations in both family-based and case-control samples [5] and two studies from different ethnic background were clearly negative in case-control [6] and both case-control and family-based designs [7]. Within European populations frequencies of allele G are consistent at 0.61 [7] and of 0.63 [present study], whereas in Asian populations allele G was the minor allele with 0.37 and 0.40 [5,6]. However, these differences hardly explain the contradictory genetic results, and assuming polygenic inheritance in schizophrenia, the negative findings could be only partially explained by sample stratification.

To replicate weak gender-specific effects at rs175174 the study sample was relatively small, but the case-control sample possesses a power of 80% to detect (at α = 0.05) an association with a susceptibility allele, under the assumption that the susceptibility allele has a population frequency of 0.63, and the effect of this allele is recessive with a relative risk of 2.5. Assuming a multiplicative model, the effect size of the female replication sample of Mukai et al. [3] is 1.6 [7]. The power against this alternative was 57% for our case-control sample of females.

The inconclusive results obtained in several genetic studies raise the question whether SNP rs175174 is itself the susceptibility allele or is in linkage disequilibrium (LD) with a further functional variant that increases risk for schizophrenia. Whereas Saito et al. [6] proposed that rs175174 is representative of ZDHHC8, differences in LD structure between the different populations may account for that the same disease causative variants yet to be identified are associated with different haplotypes. The functional variants could locate within ZDHHC8 or other genes around this locus at 22q11 and may independently or synergistically exert increased risk for schizophrenia. Therefore, the preferential transmission of individual alleles in at least a proportion of samples suggests the existence of additional functional variants in LD with SNP rs175174 or an interaction effect on the risk haplotype. In addition, the effect of the non-spliced product bearing a premature termination codon on cell homeostasis and function is still unknown [3]. On the basis of these inconsistent genetic findings on rs175174, the potential genetic diversity of the *ZDHHC8 *locus and the exact functional effect of related SNPs require further examination.

In conclusion, the findings at rs175174 at ZDHHC8 are still far from being conclusive, but evidence for a sexual dimorphism -if any- is weak. Given the large sample sizes studied together with the failure to replicate the initial findings in key respects, we agree with others [2] that the balance of evidence for ZDHHCH8 still favours the null hypothesis.

## Competing interests

The author(s) declare that they have no competing interests.

## Authors' contributions

TF carried out the molecular genetic studies and drafting of the manuscript, MG, SJ performed laboratory assays, MB participated in the design of the study and its coordination, BP, BJ participated in the recruitment of the study sample, MK performed the data-analysis, interpretation of the data, and drafting of the manuscript, GS participated in the design and coordination of the study, interpretation of the data, and drafting of the manuscript. All authors read and approved the final manuscript.

## Pre-publication history

The pre-publication history for this paper can be accessed here:


